# Over Expression of Long Non-Coding RNA PANDA Promotes Hepatocellular Carcinoma by Inhibiting Senescence Associated Inflammatory Factor IL8

**DOI:** 10.1038/s41598-017-04045-5

**Published:** 2017-06-23

**Authors:** Chuanhui Peng, Wendi Hu, Xiaoyu Weng, Rongliang Tong, Shaobing Cheng, Chaofeng Ding, Heng Xiao, Zhen Lv, Haiyang Xie, Lin Zhou, Jian Wu, Shusen Zheng

**Affiliations:** 1 Division of Hepatobiliary and Pancreatic Surgery, Department of Surgery, First Affiliated Hospital, School of Medicine, Zhejiang University, Hangzhou, China; 2 Key Laboratory of Combined Multi-organ Transplantation, Ministry of Public Health, Key Laboratory of Organ Transplantation, Zhejiang Province, Hangzhou, China; 3 Collaborative innovation center for Diagnosis treatment of infectious diseases, Hangzhou, China; 4 Department of Hepatobiliary Surgery, First Affiliated Hospital, Chongqing Medical University, Chongqing, China

## Abstract

It has been reported that long non-coding RNA PANDA was disregulated in varieties types of tumor, but its expression level and biological role in hepatocellular carcinoma (HCC) remains contradictory. We detected PANDA expression in two independent cohorts (48 HCC patients following liver transplantation and 84 HCC patients following liver resection), and found that PANDA was down-regulated in HCC. Thereafter we explored its function in cancer biology by inversing its low expression. Surprisingly, overexpression of PANDA promoted HCC proliferation and carcinogenesis *in vitro* and *in vivo*. Mechanistically, PANDA repressed transcriptional activity of senescence associated inflammatory factor IL8, which leaded to inhibition of cellular senescence. Therefore, our research help to better understand the complex role of PANDA in HCC, and suggest more thoughtful strategies should be applied before it can be treated as a potential therapeutic target.

## Introduction

Hepatocellular carcinoma (HCC) is the second leading cause of cancer death worldwide^1^. There was about 466 100 newly diagnosed HCC cases and 422 100 patients died from HCC in CHINA in 2015^2^. Although early stage HCC patients benefit from surgical intervention, such as hepatic resection and liver transplantation^3^, more than half of these patients suffered from recurrence and metastasis after operation. Even worse for advanced HCC patients, so far there is no effective systemic therapy improving overall survival more than 3 months^4^. Therefore, novel molecular biomarkers for early diagnosis, prognosis evaluation and pharmaceutical options of HCC are extremely important.

Long non-coding RNAs (lncRNAs), as majority of human transcriptome, are a class of RNAs longer than 200 nucleotides that do not encode proteins, and are involved in varieties of biological processes, including cancer biology^5–7^. Long non-coding RNA PANDA (P21 associated ncRNA DNA damage activated, hereafter termed PANDA), is a 5′-capped and polyadenylated non-spliced lncRNA, which located approximately 5 kb upstream of the CDKN1A and transcribed antisense to CDKN1A^8^. By acting as a molecular decoy^9^, PANDA interacts with transcription factor NF-YA to suppress its pro-apoptotic function during a DNA damage response^8^. Meanwhile, PANDA coordinates with scaffold-attachment-factor A (SAFA) to regulate cell senescence and proliferation^10^.

However, the expression levels and biological functions of PANDA in diverse cancers remain contradictory. It is reported that PANDA was down-regulated in non-small cell lung cancer^11^ and clear cell renal cell carcinoma^12^, but up-regulated in gastric cancer^13^ and breast cancer^14^. Controversy exits even for the same tissue type of cancer. Puvvula *et al*.^10^ reported that PANDA was significantly reduced in HCC, nevertheless Peng *et al*.^15^ uncovered that PANDA was overexpressed in HCC. These studies indicate that PANDA plays a complicated role in cancers, and its exact expression level and underlying function in HCC need to be further clearly illuminated.

In the present study, we demonstrated that PANDA was significantly lower expressed in HCC tissue, but conversely had a tumor-promoting feature both *in vivo* and *vitro*. Importantly, this aberrant function was attributed to its inhibitory action on cell senescence by suppressing senescence associated inflammatory chemokine IL8.

## Results

### Low expression of long noncoding RNA PANDA in hepatocellular carcinoma

**Figure 1:**
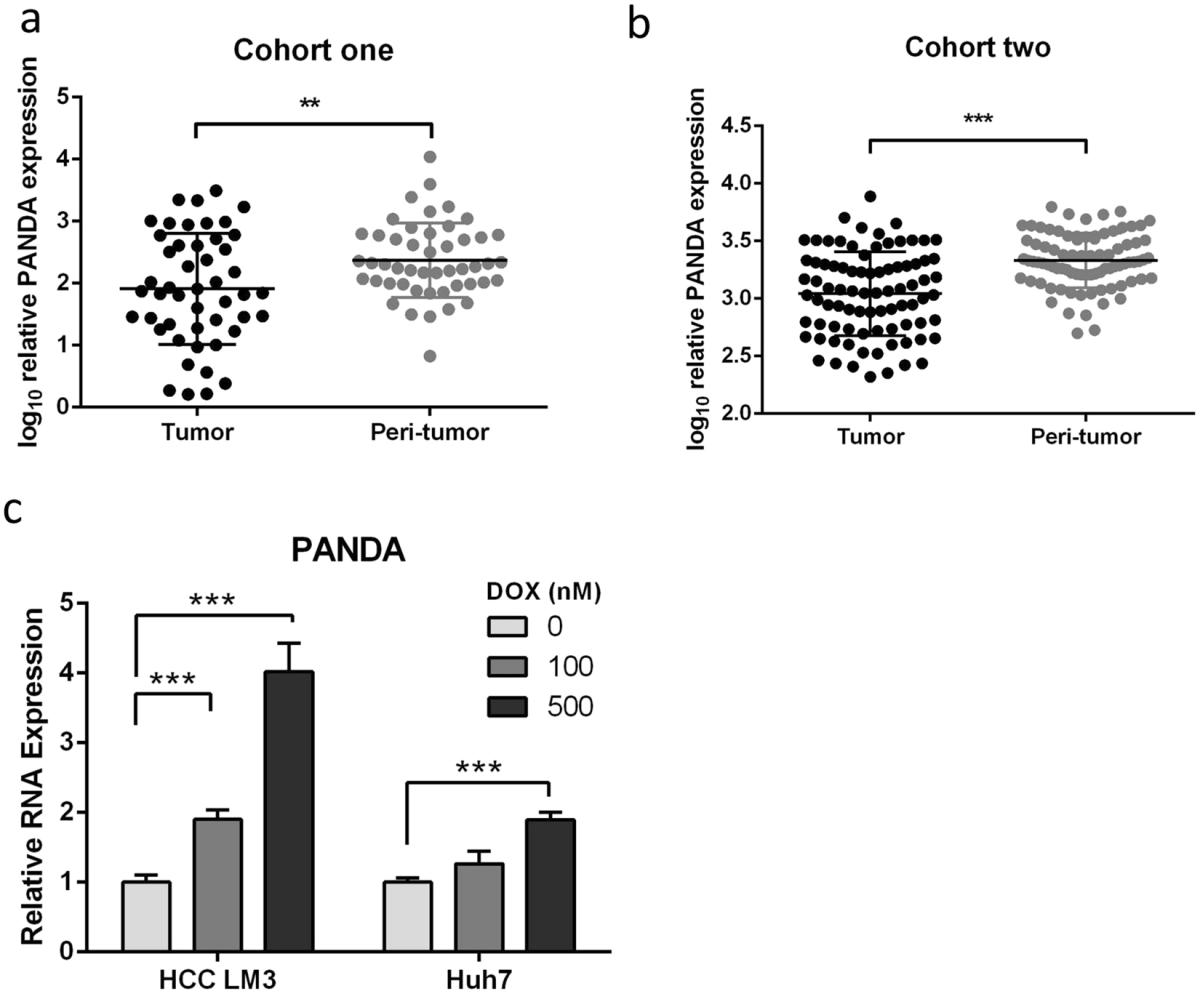
PANDA expression level in indicated samples and treatment. Long non-coding RNA PANDA was significantly lower in liver tumor tissue compared with those in peri-tumor tissue in (**a**) cohort one following liver transplantation and (**b**) cohort two following liver resection. PANDA was induced in HCC LM3 and Huh7 cell lines when these cells were treated with doxorubicin (DOX) at indicated dose for 24 hours (**c**).

The expression of long noncoding RNA PANDA in hepatocellular carcinoma (HCC) was not clear. Puvvula *et al*.^10^ reported that PANDA was low expressed in HCC compared with normal liver tissues and not associated with p53 mutational status in HCC, however, Peng *et al*.^15^ uncovered that PANDA was high expressed in HCC tissues and cell lines. Since their data were inconsistent, we employed large collections of clinical samples to further detect PANDA expression by qRT-PCR, cohort one (48 pairs of liver tumor and corresponding peri-tumor samples following liver transplantation) and cohort two (84 pairs of those following hepatic resection). We found that PANDA was significantly low expressed in tumor tissues compared with peri-tumor tissues, p < 0.01 in cohort one (Fig. 1a) and p < 0.001 in cohort two (Fig. 1b). Hung *et al*.^8^ reported that PANDA is a p53 effector. To demonstrate whether PANDA was induced by p53-mediated DNA damage in HCC, we treated two liver cancer cell lines HCC-LM3 and Huh7 with doxorubicin (DOX) at 100 nM and 500 nM for 24 hours, and found that PANDA expression was elevated and had a dose-dependent relation(Fig. 1c). Therefore, clinical investigation in our center supported that PANDA was lower expressed in HCC and induced by p53-mediated DNA damage.

### Overexpression of PANDA promotes hepatocellular carcinoma *in vitro*

**Figure 2:**
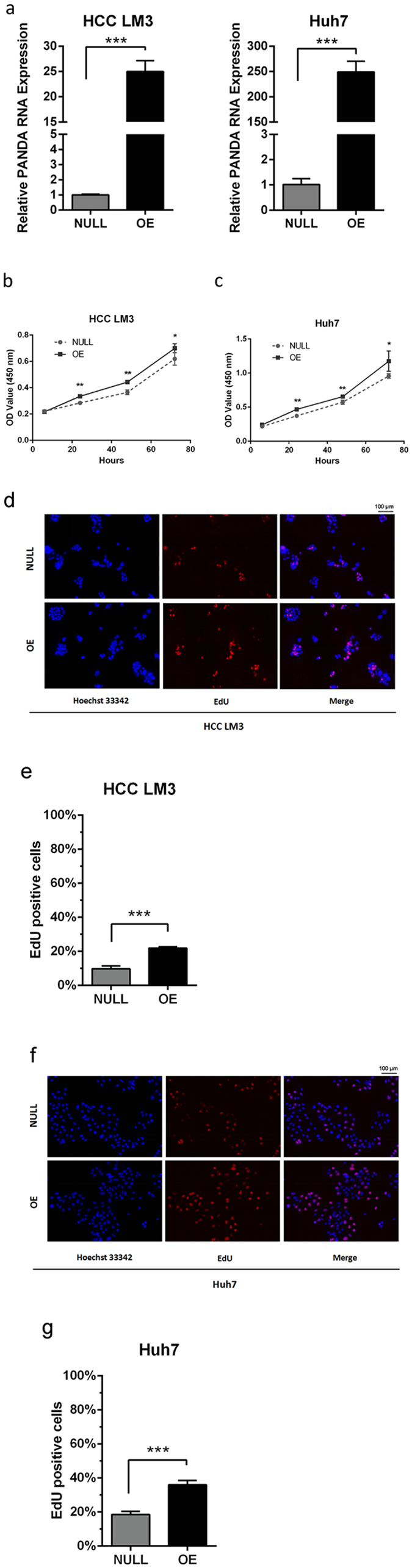
Overexpression of PANDA increases proliferating capacity of liver cancer cells *in vitro*. (**a**) Effects of overexpression of PANDA in HCC LM3 and Huh7 cells by lentivirus were verified by qRT-PCR. For CCK-8 assay, HCC LM3 (**b**) and Huh7 (**c**) cells with higher expressions of PANDA had significantly elevated OD values (450 nm) at 24, 48, 72 hours. For EdU assay, HCC LM3 (**e**) and Huh7 (**g**) cells with higher expressions of PANDA had more percentage of EdU positive cells. Representative pictures are presented in (**d**,**f**).

Since the lower expression of PANDA was detected in HCC tissues, we built two highly PANDA-expressing liver cancer cell lines, HCC LM3 and Huh7 by lentivirus (Fig. 2a). PANDA is transcribed antisense to CDKN1A (p21) and located in upstream of CDKN1A^8^, and CDKN1A functions as a regulator of cell cycle progression, therefore we set CCK-8 assay and EdU assay to evaluate PANDA’s role in cancer cell viability and proliferation. We uncovered that liver cancer cells with higher level of PANDA had an obvious tendency to proliferate. At 24, 48 and 72 hours, HCC LM3 (Fig. 2b) and Huh7 (Fig. 2c) with overexpression of PANDA showed higher OD value than control group. Moreover, HCC LM3 (Fig. 2d) and Huh7 (Fig. 2f) containing higher level of PANDA had more percentage of EdU positive cells, shown in (Fig. 2e,g) respectively. These results indicate that PANDA may have a positive biological function in tumor cell proliferation.

### Elevated expression of PANDA augments *in vivo* carcinogenesis of HCC

**Figure 3:**
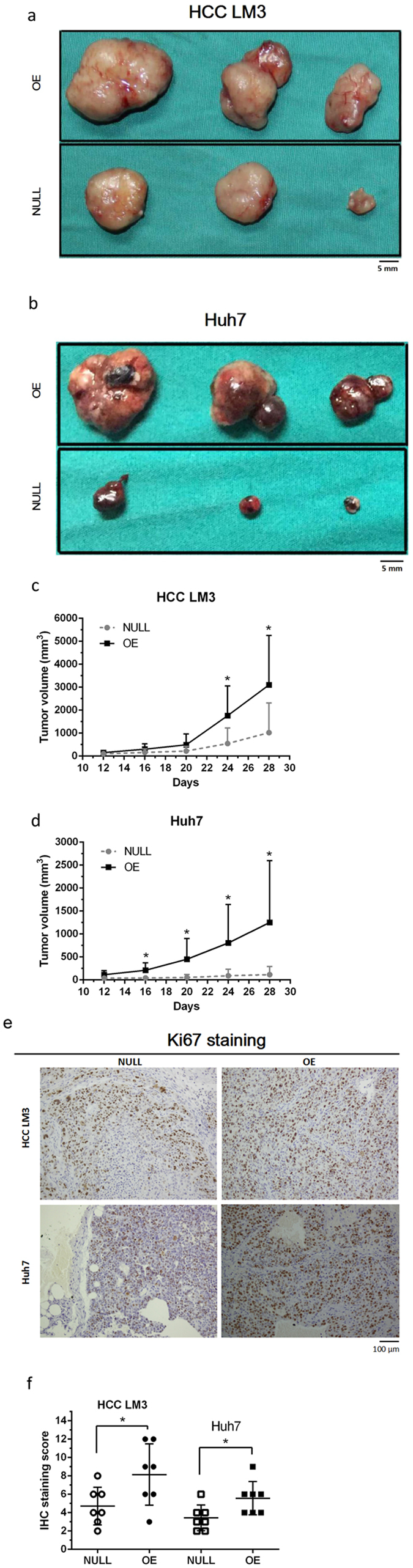
Overexpression of PANDA promotes liver cancer tumorigenesis *in vivo*. Mice bearing HCC LM3 (**c**) cells and Huh7 (**d**) cells with higher expression of PANDA (OE) had larger xenograft tumor sizes compared with control group (NULL). Representative pictures of 3 specimens from both groups were shown in (**a**,**b**) respectively. IHC staining of proliferation marker, Ki67 was consistently stronger in OE group than NULL group (**e**). IHC staining scores were analyzed in (**f**).

To further explore the promoting function of PANDA in tumor proliferation, we injected tumor cells subcutaneously into flanks of immunodeficient mice to build xenograft tumor model. Tumor sizes were measured every four days from the 12th day after implantation. Four weeks later, we sacrificed all the mice and excised tumors out. The representative pictures were shown in Fig. 3a and b. Consistently mice bearing tumor cells with higher level of PANDA had larger tumors (Fig. 3c and d). Then we detected the proliferating activities of tumor masses by staining ki67. The tumor masses generated by highly PANDA-expressing cells had more positive staining areas and stronger intensities of Ki67 (shown in Fig. 3e and analyzed in Fig. 3f). These results supported that long non-coding RNA PANDA not only promoted tumor proliferation *in vitro*, but also increased tumor growth *in vivo*.

### PANDA reinforces tumor growth by impairing senescence

**Figure 4:**
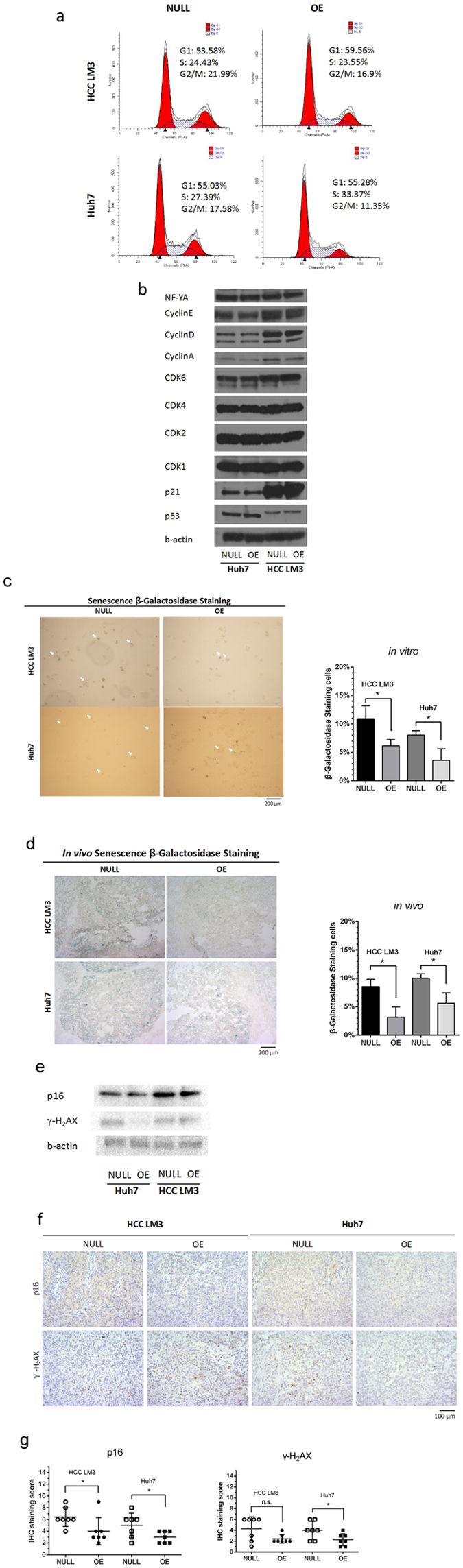
PANDA contributes to tumor growth by inhibiting senescence. (**a**) Representative pictures of cell cycle analysis show that there are no significant difference of G1/S transition between PANDA-overexpressing cells (OE) and control cells (NULL). (**b**) Cell cycle related proteins in both NULL and OE groups were detected by western blots. The corresponding full-length blots were exhibited in Supplementary Figure 1. (**c**) *In vitro* cultured cells stained with senescence associated β-galactosidase in both groups were presented and analyzed statistically. (**d**) *In vivo* xenograft tumor samples stained with senescence associated β-galactosidase in both groups were presented and analyzed statistically. (**e**) Senescence markers p16 and γ-H_2_AX were detected by western blots in indicated cells. The corresponding full-length blots were exhibited in Supplementary Figure 2. (**f**) Senescence markers p16 and γ-H_2_AX were detected by IHC in indicated xenograft tumor samples. (**g**) IHC staining scores were analyzed.

To illustrate why PANDA could promote liver cancer growth, we used flow cytometry and western blots to assess the impact of PANDA on cell cycle. Unfortunately, no significant transition of G1 phase/S phase in cell cycle has been detected in over-expressing PANDA liver cancer cells compared with control groups, as shown in Fig. 4a. Meanwhile, cell cycle promoting cyclins (Cyclin A, Cyclin D and Cyclin E) and cyclin dependent kinases (CDK1, CDK2, CDK4 and CDK6) were comparative in immunoblotting assay (Fig. 4b). Interestingly, although PANDA was a p53 effector that sensitize tumors to apoptosis by DNA damage^8^, over-expression of PANDA did not alter p53 expression without external stimulus (Fig. 4b). Moreover, p21, the transcriptional target of p53 which could cause cell cycle arrest, did not change either (Fig. 4b). In proliferating liver cancer cells, HCC LM3 and Huh7, the transcription factor NF-YA remained relatively high, and unchanged by excessive PANDA (Fig. 4b). All above results suggested that PANDA might promote tumor growth through other mechanisms rather than regulation of cell cycle and apoptosis.

It has been reported that PANDA recruited complexes to repress senescence-promoting genes^10^. Positive blue signal in β-galactosidase staining was a biomarker that well identified in senescent cells both in culture and *in vivo* ^16^. We employed β-galactosidase staining to identify senescent cells in PANDA-overexpressing groups and control groups. Even though the number of senescent cells among liver cancer cells was relatively low, there were significantly fewer blue stained cells in OE groups than NULL groups (Fig. 4c). And for xenograft tumor nodules, *in vivo* β-galactosidase staining showed similar results (Fig. 4d). Moreover, other markers of celluar senescence, such as cell cycle inhibitor p16^17^ and senescence-associated DNA damage foci characterized by phosphorylated histone H_2_AX (γ-H_2_AX)^18^, were examined in cultured cell lines and xenograft tumor samples. We also found that p16 and γ-H_2_AX were decreased while PANDA was overexpressed (Fig. 4e–g). These results indicated that overexpression of PANDA impaired senescence in tumor cells, and these tumor cells who bypassed senescence barrier contributed to tumor growth.

### Over expression of PANDA inhibits senescence associated factor IL8

**Figure 5:**
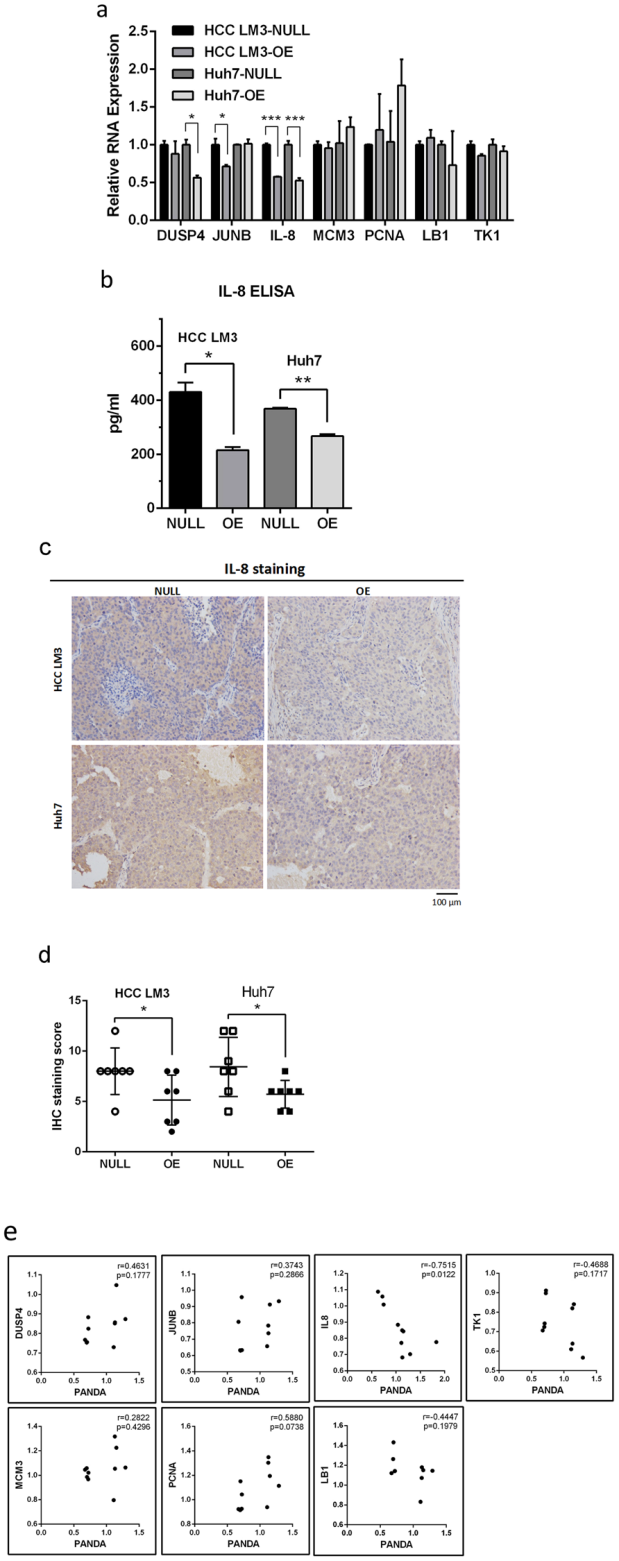
Overexpression of PANDA inhibits senescence associated factor IL8. (**a**) The transcriptional target genes of PANDA-PRC complexes were examined by qRT-PCR in both PANDA-overexpressing groups (OE) and control groups (NULL). (**b**) Cells in OE group secreted less IL8 into culture supernatant comparing with NULL group. (**c**) IL8 staining in xenograft model samples generated by OE cells were stronger than those of NULL cells. (**d**) IHC staining scores were analyzed. (**e**) The clinical relevance of PRC target genes and lncRNA PANDA were analyzed in a small number of patients in cohort two.

The molecular mechanism that lncRNA PANDA regulates cellular processes was to bind to transcription factors or RNA binding proteins, such as NF-YA or SAFA, and then to form polycomb repressive complexes (PRCs) to regulate downstream genes^8, 10^. DUSP4, JUNB, IL8, MCM3, PCNA, LB1, TK1 had been reported as PRCs’ target genes and involved in cellular proliferation and senescence^10^. In order to determine whether these target genes were modulated by PANDA in our research, we used qRT-PCR to examine their expression. Remarkably, only chemokine IL8 was restrained to half level in PANDA-overexpressing group compared with control group both in HCC LM3 and Huh7(Fig. 5a). IL8 was a typical chemokine secreted by senescent cells and induced neighboring cells to occur senescence by paracrine fashion^19^. We hypothesized that redundant PANDA inactivated the IL8 transcription, caused less IL8 secretion, thus inhibited cellular senescence. To further verify this hypothesis, we investigated IL8 secretion from cell culture supernatant using ELISA, and IL8 staining from xenograft samples using immunohistochemistry. Consistently, cells expressing higher PANDA released less IL8 into cell culture (Fig. 5b), and tumor nodules generated from highly-expressing-PANDA cells expressed lower IL8 (Fig. 5c and d). In addition, clinically we examined these PRC target genes as well as PANDA in a small number of HCC tumor samples from cohort two, and analyzed their correlation. Among all these target genes, IL8 had a significant negative relevance with PANDA (Fig. 5e). Therefore, we discovered that IL8 was downregulated by lncRNA PANDA during cellular proliferation and senescence.

### Ectopic expression of IL8 rescues PANDA-mediated phenotype

**Figure 6:**
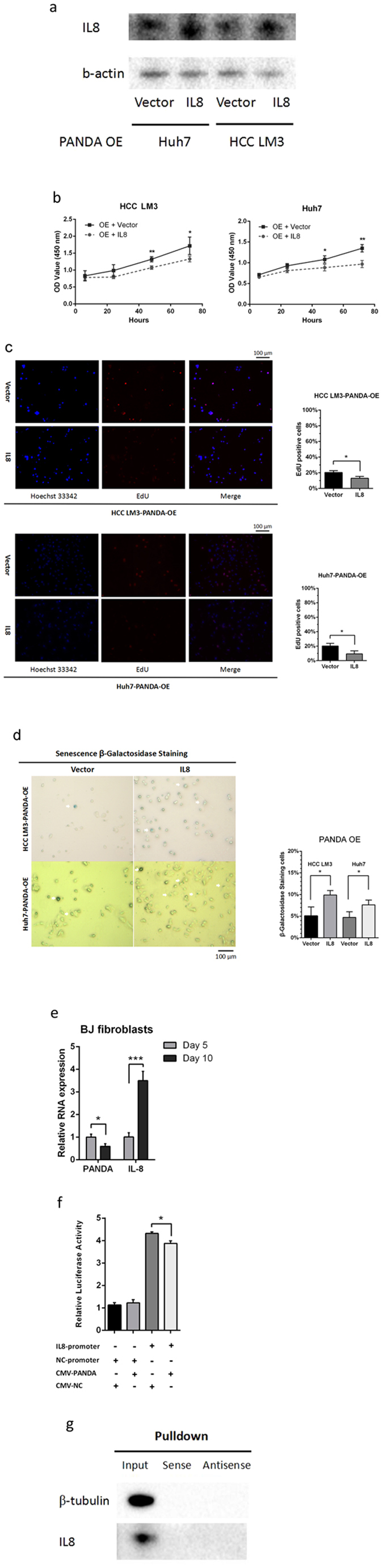
Overexpression of IL8 rescues PANDA-mediated phenotype. (**a**) Effects of overexpression of IL8 in HCC LM3-PANDA-OE and Huh7-PANDA-OE cells by plasmid were confirmed by western blots. The corresponding full-length blots were exhibited in Supplementary Figure 3. (**b**) PANDA-overexpressed cells transfected with IL8 plasmid had reduced OD values (450 nm) at 24, 48, 72 hours compared with those with empty vector. (**c**) For EdU assay, PANDA-overexpressed cells transfected with IL8 plasmid had less percentage of EdU positive cells. (**d**) For *in vitro* senescence associated β-galactosidase staining, PANDA-overexpressed cells transfected with IL8 plasmid had more blue stained cells. (**e**) RNA expression levels of PANDA and IL8 were analyzed in BJ fibroblasts at indicated time after transfected with NRas^G12V^ plasmid. (**f**) The reporter activity of IL8 promoter in 293 T with or without overexpression of PANDA was determined by dual luciferase reporter assay system. (**g**) Huh7 cell lysates were incubated with *in vitro*-transcribed biotin-labeled sense or antisense PANDA for RNA pull-down assay, followed by western blot analysis with indicated antibodies. The corresponding full-length blots were exhibited in Supplementary Figure 4.

In order to demonstrate the enhanced proliferation and bypassed senescence in PANDA-overexpressed cells is participated by IL8, we induced IL8 expression by plasmids in Huh7-PANDA-OE and HCC LM3-PANDA-OE cells (Fig. 6a). We found that overexpression of IL8 in these cells could reverse the phenotype caused by introduction of PANDA, detected by CCK-8 assay (Fig. 6b), EdU assay (Fig. 6c) and *in vitro* β-galactosidase staining (Fig. 6d). Oncogene-induced senescence was a classical model to dissect senescence^19^. We transfected BJ fibroblast cells with NRas^G12V^ plasmids, which had been used for senescence induction^20^, to further confirm that lncRNA PANDA and IL8 had a negative relevance during senescence. From the fifth day to the tenth day after transfection, IL8 increased while PANDA decreased (Fig. 6e). These results indicated that IL8 mediated the biological function of PANDA in HCC.

However, the underlying mechanism how PANDA regulated IL8 still remained unknown. We performed a dual luciferase reporter assay in 293 T cells to evaluate the reporter activity of IL8 promoter with or without overexpression of PANDA. As a result, the reporter activity of IL8 promoter decreased significantly when transfected with PANDA plasmid (Fig. 6f). To demonstrate whether lncRNA PANDA directly bound to IL8 protein, we conducted RNA pull down assay. Unfortunately, no evidence of direct binding of lncRNA PANDA and protein IL8 could be observed (Fig. 6g). Therefore, lncRNA PANDA might inhibit IL8 expression by forming a polycomb repressive complex targeting IL8 promoter, rather than directly binding to IL8 protein.

## Discussion

Recently, long non-coding RNA PANDA has been examined in more and more types of cancers, but its expression level and biological function has tissue specific property and remains controversial^10–15^. The inconsistent results of PANDA expression in HCC between Puvvula *et al*.’s research^10^ and Peng *et al*.’s research^15^ attracted our considerable interests. In our study, PANDA was significantly reduced in two independent cohorts of HCC patient samples. And PANDA was first reported in the literature as a effector of tumor suppressor p53, and loss of PANDA made cells more sensitive to apoptosis^8^. Therefore, in combination with our results and literature reviews, we strongly supported that PANDA was down-regulated in HCC. However, PANDA expression pattern in HCC still needs to be more clearly defined by multicentre evaluation and validation, especially according to the p53 status.

Unexpectedly, we observed a tumor promoting function of PANDA in HCC through the methods of overexpressing PANDA by lentivirus. PANDA not only increased tumor cell proliferation in cell culture but also accelerated carcinogenesis in xenograft tumor model. Oncogenes or tumor suppressors played unexpected tumor-suppressing or tumor-promoting roles in hepatocarcinogenesis had driven increasing attention recently^21^. He *et al*.^22^, Bard-Chapeau *et al*.^23^. and Wang *et al*.^24^. had explained that the conflicting phenotypes may be caused by liver compensatory proliferation due to alteration of inflammatory microenvironment. These inspired us to search underlying mechanism, not limited to cell cycle transition and apoptosis. Interestingly, we uncovered that the cellular senescence of liver cancer cells was inhibited by elevated level of PANDA.

Cellular senescence is a biological process that cell responses to diverse stress, including dysfunctional telomeres, DNA damage and strong mitogenic signals, thus acquires a long term state of proliferate arrest^25^. Senescent cells can drive degenerative and hyperplastic pathology, the most deadly of the latter is cancer^26^. Senescence-to-immortality transition has been revealed in hepatocytes, which is mediated by genome-wide transcriptional modifications involved with long non-coding RNAs^27^. One hallmark feature of senescence is that senescent cells express and secrete cytokines and growth factors to increase the complexity of tumor microenvironment, which is termed senescence-associated secretory phenotype (SASP), including IL-6, IL-8^28^. These senescence associated factors have considerable influence on neighbouring cells through paracrine fashion to apply beneficial or detrimental effects^19^.

PANDA inhibits senescence-promoting genes in proliferating cancerous cells by transcriptional interference^10^. We detected PANDA target genes and found that IL8 was significantly reduced when PANDA is overexpressed. The senescence associated inflammatory mediator, IL8 was produced when transcription factor NF-κB was activated, and its expression can be promoted by protein kinase D1 (PKD1)^29^ and suppressed by microRNAs miR-146a/b^30^. Induction of IL8 occurs in cells undergoing senescence, while repression of IL8 delays replicative senescence in outgrowth endothelial cells^31^. Here, we demonstrated that IL8 was suppressed by PANDA in HCC, thereafter inhibited cellular senescence, which allowed pre-senescent cells to escape senescence, and continue to proliferate, thus contribute to tumor promotion.

The molecular mechanism that employed by lncRNAs to regulate cellular processes has been well stated^32^. Based on the fact that the cellular location of lncRNA PANDA was nuclear, PANDA functionally participated in chromatin interactions and transcriptional regulation^7^. It is in agreement with our findings. We revealed that overexpression of PANDA directly decrease the transcription activity of IL8 promoter, but not directly binding to IL8 protein. However, how the transcription activity of IL8 promoter were suppressed by PANDA, through facilitating histone modification or serving as a assembling scaffold, remains undiscovered.

In summary, we expanded our knowledge about long non-coding RNA PANDA in HCC that PANDA was downregulated in HCC but promoted liver cancer tumorigenesis by inhibiting cellular senescence via senescence associated inflammatory mediator IL8. Our findings provide a profound understanding about the complicated role of long non-coding RNA PANDA in liver cancer, and emphasize that precise molecular mechanisms should be elucidated before targeting it as diagnostic and therapeutic agents. Moreover, we strongly suggest that genetically engineered animal model or different p53 status model should be required to investigate the biological function of lncRNA PANDA in cancer.

## Methods

### Patients and samples

48 frozen HCC tissues and the corresponding peri-tumor tissues were obtained from patients undergoing liver transplantation at the First Affiliated Hospital, School of Medicine, Zhejiang University (Hangzhou, China) between 2011 and 2012 (cohort one). Additional 84 HCC tissues were collected from patients following liver resection at the First Affiliated Hospital, School of Medicine, Zhejiang University between 2010 and 2013 (cohort two). Informed consents have been obtained from all patients. And the research was conducted with the approval of Institutional Review Board and Ethics Committee of First Affiliated Hospital, School of Medicine, Zhejiang University, and in accordance with Declaration of Helsinki.

### RNA extraction and qRT-PCR analysis

Total RNA from the specimens and cells was isolated using Trizol reagent (Invitrogen, Carlsbad, California, USA). Reverse Transcription was performed using High Capacity cDNA Reverse Transcription Kit (Applied Biosystems, Waltham, Massachusetts, USA) and qRT-PCR was performed with SYBR® Green dye (Takara, Shiga, Japan). All reactions were performed in triplicate and GAPDH RNA levels were used for normalization. The primer sequences were provided in Supplementary Table S1.

### Cell culture

Hepatocellular carcinoma cell lines, HCC LM3 and Huh7, were purchased from China Center for Type Culture Collection (CCTCC), and grown in MEM supplemented with 10% fetal bovine serum (FBS) and penicillin/streptomycin, at 37°C under an atmosphere containing 5% CO_2_. For doxorubicin (DOX, Sigma D1515, St. Louis, Missouri, USA) treatment, DOX was added to culture medium at 0 nM, 100 nM, 500 nM and maintained for 24 hours.

### Overexpression of PANDA and IL8

The lentiviral PANDA construct (OE) and empty construct (NULL) were completed by Genechem Company (Shanghai, China). Full-length PANDA (NR_109836) was gene synthesized, sequence-verified and cloned into GV341 (Ubi-MCS-3FLAG-SV40-puromycin) vector (Genechem, Shanghai, China). Lentiviral infection was performed by standard procedures. The IL8 plasmid and empty vector were purchased from Genechem Company (Shanghai, China). Transfection was performed with Lipofectamine 2000 (Invitrogen, Carlsbad, CA, USA) following the manufacturer’s instructions.

### Cell Proliferation Assay

5 × 10^3^ cells were seeded into each well of 96-well plate to perform Cell Counting Kit-8 (CCK-8) proliferation assay (Dojindo, Kumamoto, Japan) at indicated time according to the manufacturer’s instructions as previously described^33^. 2 × 10^4^ cells were seeded into Nunc™ Glass Bottom Dishes (Thermo Scientific, 150682, Waltham, Massachusetts, USA) to complete EdU imaging assay (RiboBio, C10310, Guangzhou, China) after 24 hours according to the manufacturer’s instructions.

### Immunoblotting analysis and antibodies

Protein extraction and immunoblotting analysis were performed according to the regular procedure. The following antibodies were used: NF-YA (Santa Cruz, sc-10779, Dallas, Texas, USA), CDK1 (CST, #9116, Danvers, Massachusetts, USA), CDK2 (CST, #2546), CDK4 (CST, #12790), CDK6 (CST, #3136), Cyclin A (CST, #4656), Cyclin D (CST, #2978), Cyclin E (CST, #4129), p53 (Santa Cruz, sc-126), p21 (CST, #2947), b-actin (CST, #8457), β-tubulin (CST, #2128), p16 (proteintech, 10883-1-AP), γ-H_2_AX (abcam, ab81299) and IL8 (ABGENT, AP8612B, San Diego, California, USA). The original full-length blots were exhibited in the Supplementary Information.

### Xenograft model and immunohistochemistry

5 × 10^6^ HCC LM3 and Huh7 cells expressing control (NULL) or PANDA (OE) were suspended in 0.2 ml PBS and injected subcutaneously into flanks of 8 weeks old immunodeficient mice. Tumor volumes were measured every four days from the 12th day after injection, and calculated by the formula V = ½ (length × width^2^). All the mice were sacrificed 4 weeks later after injection and tumors were harvested for immunostaining. Animal study protocols were performed in accordance with the National Institute Guide for the care and use of laboratory animals.

Immunohistochemistry and semiquantitative evaluation were performed as described previously^24^. Antibodies recognizing Ki67 (abcam, ab15580, Cambridge, UK), IL8 (ABGENT, AP8612B, San Diego, California, USA), p16 (proteintech, 10883-1-AP, Wuhan, P.R.C.) and γ-H_2_AX (abcam, ab81299, Cambridge, UK) were used in immunostaining.

### Senescence analysis

Senescence was assessed by β-galactosidase cell staining according to manufacturer’s instructions (CST, #9860). For *in vitro* assay, 1 × 10^4^ cells were seeded into Nunc™ Glass Bottom Dishes (Thermo Scientific, 150682, Waltham, Massachusetts, USA) and then followed by standard procedure. For *in vivo* assay, xenograft tumors were fixed with 0.2% PFA/0.1 M PIPES/2 mM MgCl_2_/5 mM EGTA (pH = 6.0) before freezing and then followed by standard procedure.

### Cell cycle analysis

Cells were stained with propidium iodide (BD Biosciences, San Jose, California, USA), after 75% ethanol fixation overnight, and analyzed for DNA content by flow cytometry (BD Biosciences) according to manufacturer’s instructions. The data were analyzed using ModFit LT^TM^ (Verity Software, Topsham, Maine, USA).

### ELISA

3 × 10^5^ cells were seeded into each well of 6-well plate. After serum starvation for 24 hours, cell culture supertatant was collected for detection of IL8 by Human IL-8 Platinum ELISA (BMS204/3, eBioscience, San Diego, California, USA) according to manufacturer’s instructions.

### Luciferase report assay

The 2 kb IL8 promoter upstream of the starting codon ATG was synthesized and cloned into pGL3-basic plasmid by Genechem Company (Shanghai, China). The reporter activities were measured 48 hours after co-transfection using the Dual-Luciferase Reporter Assay System (Promega, E1910). After normalization, the ratios of Firefly/Renilla luminescence were presented.

### RNA pull down assay

*In vitro* transcription was performed by using T7 RNA polymerase (Roche, 10881767001), SP6 RNA polymerase (Roche, 10810274001) and Biotin RNA Labeling Mix (Roche, 11685597910) according to the instructions provided by the manufacturer. 3 ug *in vitro*-transcribed biotin-labeled PANDA was incubated with 1 mg Huh7 lysates for 1 hour at 4 °C. Streptavidin-coupled Dynabeads M-280 (Invitrogen, 11205D) were then added to the reaction mix to isolate the RNA-protein complex.

### Statistical analysis

Statistical analyses were performed using Microsoft Office Excel and GraphPad Prism 6. Data were presented as the mean ± SD, and differences between groups were evaluated by two-tailed independent Student’s t-test. Relevance analysis was performed with Pearson’s correlation. P value less than 0.5 was recognized as statistically significant. All experiments were performed at least three replicates or three independent assays. *p < 0.05. **p < 0.01. ***p < 0.001.

## Electronic supplementary material

Supplementary Information
