# Supplementary material for: Over Expression of Long Non-Coding RNA PANDA Promotes Hepatocellular Carcinoma by Inhibiting Senescence Associated Inflammatory Factor IL8
Source: Sci Rep. 2017 Jun 23;7:4186. doi: 10.1038/s41598-017-04045-5 (PMC5482898; doi:10.1038/s41598-017-04045-5)
Supplement: Supplementary file 1 — Supplementary Information [file 41598_2017_4045_MOESM1_ESM.pdf]

Title:

Over Expression of Long Non-Coding RNA PANDA Promotes Hepatocellular Carcinoma by Inhibiting Senescence Associated Inflammatory Factor IL8

Author:

Chuanhui Peng[1,#], Wendi Hu[1,#], Xiaoyu Weng[1], Rongliang Tong[1], Shaobing Cheng[1], Chaofeng Ding[1], Heng Xiao[2], Zhen Lv[1], Haiyang Xie[1], Lin Zhou[1], Jian Wu[1,*], Shusen Zheng[1,*]

[#] These authors contributed equally to this work.

[*] Correspondence to: Dr. Jian Wu, E-mail: drwujian@hotmail.com and Prof. Shusen Zheng , E-mail: shusenzheng@zju.edu.cn

[1] Division of Hepatobiliary and Pancreatic Surgery, Department of Surgery

First Affiliated Hospital, School of Medicine, Zhejiang University,

Key Laboratory of Combined Multi-organ Transplantation, Ministry of Public Health

Key Laboratory of Organ Transplantation, Zhejiang Province, Hangzhou 310003,China

Collaborative innovation center for Diagnosis treatment of infectious diseases

[2] Department of Hepatobiliary Surgery, First Affiliated Hospital, Chongqing Medical University, Chongqing, China

Supplementary Information

Supplementary Table 1
Supplementary Figure 1
Supplementary Figure 2
Supplementary Figure 3
Supplementary Figure 4

Supplementary Table 1

qRT-PCR primer sequences		
Gene	Forward	Reverse
GAPDH	AGCCACATCGCTCAGACAC	GCCCAATACGACCAAATCC
PANDA	TGCACACATTTAACCCGAAG	CCCCAAAGCTACATCTATGACA
DUSP4	CCTGGCAGCCATCCCACCCCCGGTTCCCC	GCTGATGCCCAGGGCGTCCAGCATGTCTCTC
JUNB	ATGGAACAGCCCTTCTACCACG	AGGCTCGGTTTCAGGAGTTTG
IL8	GGCACAAACTTTCAGAGACAG	ACACAGAGCTGCAGAAATCAGG
MCM3	CGAGACCTAGAAAATGGCAGCC	GCAGTGCAAAGCACATACCGCA
PCNA	CAAGTAATGTCGATAAAGAGGAGG	GTGTCACCGTTGAAGAGAGTGG
LB1	CCGTTGACCAGCGAAGACATAC	TTAGCACTCTTCCTTTGCCAGCT
TK1	AGCAGCTTCTGCACACATGACC	CTCGCAGAACTCCACGATGTCA

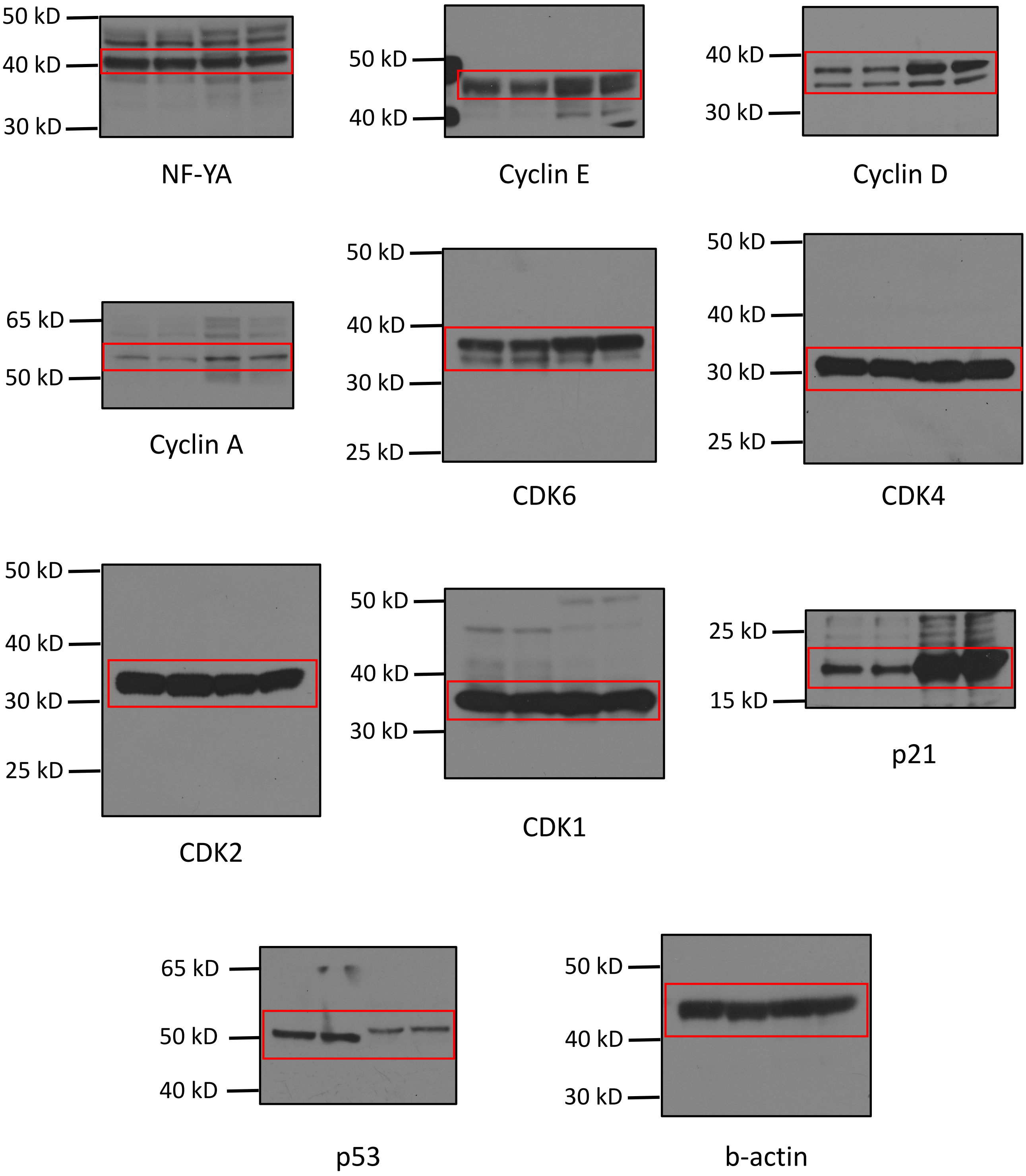

Supplementary Figure 1:

Full-length blots corresponding to Fig.4b. Indicated parts (red box) are shown in Fig.4b.

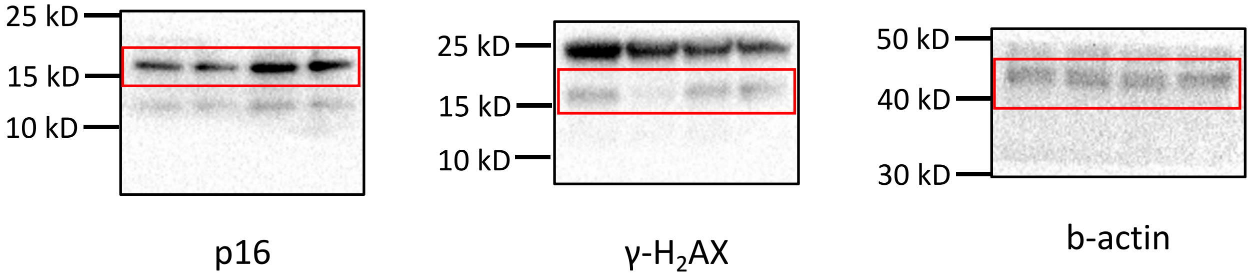

Supplementary Figure 2:

Full-length blots corresponding to Fig.4e. Indicated parts (red box) are shown in Fig.4e.

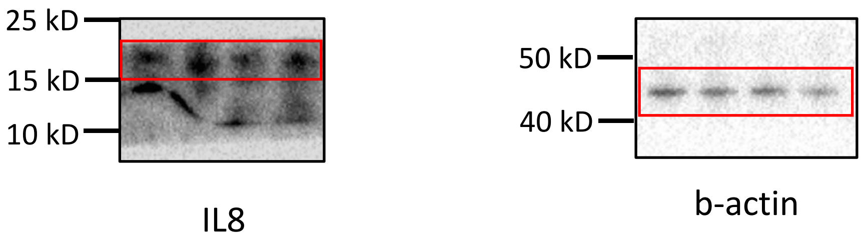

Supplementary Figure 3:

Full-length blots corresponding to Fig.6a. Indicated parts (red box) are shown in Fig.6a.

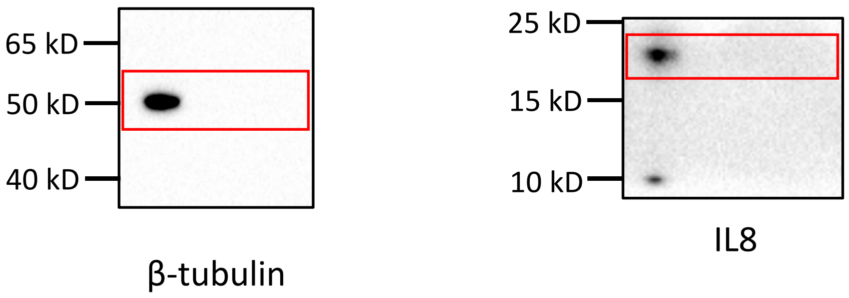

Supplementary Figure 4:

Full-length blots corresponding to Fig.6g. Indicated parts (red box) are shown in Fig.6g.
